# The Impact of Calcium and Vitamin D Supplementation Prior to Thyroidectomy on Mitigating Postoperative Hypocalcemia

**DOI:** 10.7759/cureus.51709

**Published:** 2024-01-05

**Authors:** Maryam Riaz, Ismail Akbar, Maria Qureshi, Rao E Hassan, Habib Ur Rehman, Asna Tahir, Muhammad Daud, Kainat Bibi, Shafiq Tanveer

**Affiliations:** 1 Department of Surgery, Ayub Teaching Hospital, Abbottabad, PAK; 2 Department of Family Medicine, Ayub Teaching Hospital, Abbottabad, PAK; 3 General Surgery, Hayatabad Medical Complex Medical Teaching Institute (MTI), Peshawar, PAK; 4 Ophthalmology, Khyber Teaching Hospital Medical Teaching Institute (MTI), Peshawar, PAK; 5 Surgery, Lady Reading Hospital Medical Teaching Institute (MTI), Peshawar, PAK; 6 Department of Internal Medicine, Ayub Teaching Hospital, Abbottabad, PAK

**Keywords:** quasi-experimental study, vitamin d, serum calcium, thyroidectomy, postoperative hypocalcemia

## Abstract

Background

Hypocalcemia remains the most frequent complication after thyroidectomy. It can either be transient or permanent, and patients often find it unpleasant due to its association with prolonged hospitalization. The objective of this study was to determine the role of preoperative calcium and vitamin D supplementation in preventing hypocalcemia after subtotal/total thyroidectomy.

Material and methods

This quasi-experimental study was conducted at the Department of General Surgery, Ayub Teaching Hospital, Abbottabad, Pakistan, from January 2023 to July 2023. We utilized non-probability purposive sampling. Patients undergoing total thyroidectomy were recruited and followed until discharge to ascertain outcomes. They were divided into two groups: Group A received vitamin D (200,000 IU) one week preoperatively as single intramuscular dose and calcium (1 gm) orally two times daily for one week preoperatively, while Group B served as the control. Venous blood samples were taken post-surgery, on the second and seventh day and at day 30 to assess hypocalcemia. Chi-square test was employed, comparing hypocalcemia in both groups with a p-value ≤0.05 considered significant.

Results

One hundred thirty-two patients underwent subtotal/total thyroidectomy, with 46.2% (n = 61) being male and 53.8% (n = 71) female. The mean age of these patients was 37.12 ± 6.22 years, ranging from 20 to 50 years, and 57.6% (n = 76) were aged over 35 years. More than half (55.3%, n = 73) hailed from rural areas, while 4.7% (n = 59) resided in urban locales. Among the patients, 15.9% (n = 21) had a history of diabetes, and 24.2% (n = 32) were hypertensive. The mean body mass index (BMI) was 23.32 ± 2.41 kg/m^2^, with 7.6% (n = 10) classified as obese. The mean preoperative serum calcium level was 9.87 ± 1.07 mg/dL. Postoperatively, the mean serum calcium level was 8.74 ± 0.83 mg/dL. Both Group A (preoperative vitamin D and calcium supplementation) and Group B (control) demonstrated comparable baseline characteristics before undergoing thyroidectomy. The incidence of postoperative hypocalcemia was notably lower in Group A, with only 4.5% (n = 3) experiencing this complication. By contrast, 24.2% (n = 16) of patients in Group B, the control group, developed hypocalcemia (P = 0.001).

Conclusion

Our study supports the use of preoperative calcium and vitamin D supplementation in patients undergoing thyroidectomy to combat hypocalcemia. The treated group showed significantly lower hypocalcemia compared to the untreated group B. We recommend preoperative calcium and vitamin D supplementation for all thyroidectomy patients to reduce related morbidities and hospitalization duration.

## Introduction

The global prevalence of thyroid diseases is increasing, impacting 2% to 8% of the population, particularly in iodine-deficient regions [1,2]. Notably, hyperthyroidism is more common in women, affecting 0.5% to 2%, whereas men experience it at a rate 10 times lower. Overt hypothyroidism is reported at approximately two to four cases per 1,000 population per year, with a prevalence ranging from 0.8% to 1.3% [3].

Total thyroidectomy, a surgical intervention for various thyroid diseases, involves the removal of thyroid glands. Despite associated risks, this procedure is performed to mitigate recurrence risks, especially in benign cases, underscoring the importance of understanding the clinical profiles of thyroidectomy cases for public health [4].

Thyroid surgery, whether it be total, near-total, or subtotal, comes with inherent risks, including severe complications, such as vocal cord paralysis and significant bleeding [5]. Despite these potential challenges, the most frequently encountered post-surgery complication is hypocalcemia. Transient hypocalcemia frequently occurs during the postoperative period of the surgery among patients who have recently undergone thyroid surgery [6]. Hypocalcemia after thyroidectomy results from removal, devascularization, and damage of the parathyroid glands, which can lead to transient or permanent hypoparathyroidism [7]. Recent studies reveal varying rates of postoperative hypocalcemia after total thyroidectomy, with Malik et al. reporting a 54% incidence [8], Alhefdi et al. documenting 4.6% versus 20.5% hypocalcemia in untreated versus treated patients in the USA [9], and Sajid et al. reporting a 35% incidence in cases undergoing total thyroidectomy [10].

This study aims to evaluate the burden of postoperative hypocalcemia in cases undergoing total/subtotal thyroidectomy, treated with preoperative calcium and vitamin D supplementation. Early diagnosis and timely management are crucial for correcting low serum calcium levels, highlighting the necessity of calcium supplementation to achieve desired clinical outcomes and enhance the quality of life for these patients.

## Materials and methods

This quasi-experimental study was conducted at the Department of General Surgery, Ayub Teaching Hospital in Abbottabad, Pakistan, spanning from January to July 2023. Approval for the study (approval number: RC-EA-2023/011) was granted by the Medical Teaching Institute Abbottabad Institutional Ethical Review Committee, and participants provided written informed consent for participation in the study. Utilizing a non-probability purposive sampling technique, the study established a sample size of 132 (66 in each group) using the World Health Organization software for sample size calculation, with a 95% confidence interval, considering hypocalcemia rates of 26.1% versus 6.5% in postoperative untreated and treated patients [8].

Inclusion criteria encompassed individuals aged 18-50 of either gender undergoing subtotal/total thyroidectomy, while exclusions included those with preoperative hypocalcemia, a history of thyroid surgeries, tumors, heart diseases, chronic renal or liver conditions, and traumatic injuries. Baseline data, covering gender, age, obesity, diabetes, hypertension, and residential status, were documented at the time of registration.

Enrolled patients undergoing subtotal/total thyroidectomy were randomly assigned to two groups. Group A (66 patients) received a preoperative regimen of vitamin D (200,000 IU) administered as a single intramuscular dose and oral calcium carbonate (1 gm) twice daily for one week before the surgery. Group B (66 patients) served as the control. Thyroidectomy procedures adhered to hospital protocols, and blood samples were collected post-surgery, on days two, seven, and 30 for the analysis of serum calcium levels. Hypocalcemia was defined as a postoperative serum calcium level below 8.0 mg/dL in any of these four sampling occasions.

Data collection and analysis were performed using IBM SPSS Statistics for Windows, version 24.0 (released 2021; IBM Corp., Armonk, New York, United States). Qualitative variables, such as gender, diabetes, hypertension, residential status, and hypocalcemia, were presented as frequencies and percentages. Quantitative variables, including body mass index (BMI), age, and calcium level, were expressed as mean ± standard deviation (SD). The chi-square test was employed to compare the frequency of hypocalcemia between the two groups, with a p-value ≤0.05 considered significant (95% confidence interval).

## Results

One hundred thirty-two patients underwent subtotal/total thyroidectomy in our study. Among them, 46.2% (n = 61) were male, and 53.8% (n = 71) were female. The mean age of these patients was 37.12 ± 6.22 years, ranging from 20 to 50 years, with 57.6% (n = 76) being over 35 years old. Of the participants, 55.3% (n = 73) hailed from rural areas, while 4.7% (n = 59) resided in urban areas. Notably, 15.9% (n = 21) had a history of diabetes, and 24.2% (n = 32) were hypertensive. The mean BMI was 23.32 ± 2.41 kg/m^2^, and 7.6% (n = 10) who had the BMI of >30.0 were classified as obese. The mean preoperative serum calcium level was 9.87 ± 1.07 mg/dL. Following the surgery, the mean postoperative serum calcium level was 8.74 ± 0.83 mg/dL. Both groups were comparable in terms of baseline characteristics before undergoing thyroidectomy (Table 1).

**Table 1:** Baseline characteristics in both groups Chi-square test used with no p-value ≤0.05, demonstrating no significant difference between the baseline characteristics of the two groups.

Characteristics	Groups		P value
	Group A (n = 66)	Group B (n = 66)	
Gender			
Male (n = 61)	28 (42.4%)	33 (50.0 %)	0.383
Female (n = 71)	38 (57.6%)	33 (50.0 %)	
Age groups			
Up to 35 years (n = 56)	24 (36.4 %)	32 (48.5 %)	0.159
>35 years (n = 76)	42 (63.6 %)	34 (51.5 %)	
Residential status			
Rural (n = 73)	35 (53.0 %)	38 (57.6 %)	0.599
Urban (n = 59)	31 (47.0 %)	28 (42.4 %)	
Diabetes			
Yes (n = 21)	14 (21.2%)	07 (10.6 %)	0.096
No (n = 111)	52 (78.8%)	59 (89.4 %)	
Hypertension			
Yes (n = 32)	20 (30.3%)	12 (18.2 %)	0.104
No (n = 100)	46 (69.7%)	54 (81.8 %)	
Obesity			
Yes (n = 10)	06 (9.1 %)	04 (6.1%)	0.511
No (n = 122)	60 (90.9 %)	62 (93.9 %)	

Postoperative hypocalcemia was observed in 4.5% (n = 3) of patients in Group A and 24.2% (n = 16) in Group B, indicating a significant difference (p = 0.001) (Table 2).

**Table 2:** Distribution of postoperative hypocalcemia in both groups. Chi-square test used with a p-value ≤0.05, demonstrating significant difference between both groups.

Hypocalcemia	Group A		Group B	
	Frequency	Percentage	Frequency	Percentage
Yes (n = 19)	03	4.5	16	24.2
No (n = 113)	63	95.5	50	75.8
P value	0.001			

## Discussion

Thyroid surgeries are performed globally, and total thyroidectomy has become the preferred approach for managing the majority of thyroid diseases [11]. However, a common complication associated with this procedure is transient hypocalcemia, affecting approximately 24% of cases [12]. This complication not only increases morbidity but also extends the duration of hospitalization [13]. Transient hypocalcemia becomes a significant concern, especially after total thyroidectomy, often leading to prolonged hospital stays or subsequent readmissions. While bleeding stands out as the primary complication in the initial 24 hours post-surgery, transient hypocalcemia becomes the primary concern from the second day up to six months following the procedure. The assessment of low serum calcium levels can be conducted through symptomatic evaluation or laboratory testing. Common signs and symptoms of hypocalcemia include numbness, tingling sensations, and carpopedal spasms [14]. The administration of oral calcium and vitamin D, both before and after surgery, has demonstrated efficacy in preventing the onset of postoperative transient hypocalcemia following thyroidectomy [15].

Our study comprised 132 patients, with 46.2% (n = 61) being male and 53.8% (n = 71) female. Notably, findings from various studies align with our gender distribution. Malik et al. reported 52.2% female patients undergoing thyroidectomy [8], while Essa et al. in Egypt noted 77% female patients [16]. Similarly, studies by Qi et al. [17] and Li et al. [18] from China, as well as Sittitrai et al. [19], indicated a predominance of female patients, with percentages ranging from 74.5% to 89%. Baloch et al. conducted a substantial study involving 854 patients, revealing 78% female patients undergoing thyroidectomy [20]. In a study by Althoubaity et al. from King Abdulaziz University Hospital, Kingdom of Saudi Arabia, 69.5% of patients undergoing total thyroidectomy were female [21]. These consistent findings across diverse studies underscore a notable predominance of female patients undergoing thyroidectomy.

The mean age of patients undergoing thyroidectomy was 37.12 ± 6.22 years, with 57.6% (n = 76) aged over 35 years. Comparable mean ages have been reported in related studies. Malik et al. documented a mean age of 38.67 ± 8.63 years in their study on patients undergoing thyroidectomy [8]. Similarly, Essa et al. observed a mean age of 48.17 ± 6.54 years in their study population [16]. Qi et al. reported a mean age of 46.4 ± 5.1 years for patients undergoing thyroidectomy [17], while Li et al. found a mean age of 41 years in their study [18]. Sittitrai et al. noted a mean age of 47.2 ± 13.6 years among patients undergoing thyroidectomy [19], and Baloch et al. reported a mean age of 42.1 ± 14.76 years (range: 14-76 years) [20]. In a study by Althoubaity et al. involving 154 patients, the mean age of those undergoing total thyroidectomy was 43.23 ± 12.16 years [21]. These consistent findings across studies indicate a notable incidence of older patients undergoing thyroidectomy.

Out of all the participants, 73 individuals (comprising 55.3%) hailed from rural regions, while a smaller percentage, 4.7% (n = 59), resided in urban areas. This distributional difference contrasts with the findings of Qi et al., who reported that 68% of individuals undergoing thyroidectomy were from urban areas [17]. The variance may be attributed to the extensive coverage of large peripheries by our institution.

A history of diabetes was noted in 15.9% (n = 21), and 24.2% (n = 32) of the participants were hypertensive. Essa et al. reported slightly higher prevalence rates for diabetes (31%) and hypertension (42%) compared to our study. The mean BMI was 23.32 ± 2.41 kg/m², with 7.6% (n = 10) classified as obese [16]. In a similar context, Qi et al. presented a mean BMI of 23.8 ± 3.3 kg/m² for individuals undergoing thyroidectomy [17].

The average preoperative serum calcium level was 9.87 ± 1.07 mg/dL, and the postoperative level was 8.74 ± 0.83  mg/dL. In the study by Malik et al., they reported a similar mean preoperative serum calcium level of 9.48 ± 0.49 mg/dL, aligning closely with our results [8]. Comparable findings were also observed in the study by Li et al. [18]. Althoubaity et al. conducted a study that offered significant insights into calcium level assessments on consecutive days following surgery. Notably, hypocalcemia reached its peak on the second day, affecting 87 patients, constituting 67.4% of the total sample. The average calcium level measured on the second day was 2.03±0.17 mmol/L, falling below the normal range of 2.12 mmol/L-2.52 mmol/L [21].

In our investigation, postoperative hypocalcemia was observed in 4.5% (n = 3) of participants in group A and 24.2% (n = 16) in group B (p = 0.001). Similarly, Malik et al. reported hypocalcemia rates of 6.5% in the treated group and 26.1% in the untreated group, mirroring our study outcomes [8]. In addition, Li et al. found a significant association between preoperative supplementation and a reduced incidence of hypocalcemia [18]. Sittitrai et al. reported hypocalcemia in 8.8% compared to 22.7% of individuals undergoing thyroidectomy, supporting our results [19]. This suggests that our research findings align with those of analogous studies in the literature, underscoring their significance.

The study has notable limitations. These include a relatively small sample size of 132 patients, a confined age range (18-50 years), and specific inclusion/exclusion criteria that limit the generalizability of findings. In addition, short-term outcomes focused solely on the first 30 days post-surgery, neglecting potential long-term effects. The single-center nature of the study may further limit external validity. These limitations collectively underscore the importance of exercising caution when interpreting the results. They highlight the necessity for future research with larger, more diverse samples and extended follow-up periods to comprehensively understand the efficacy of the studied interventions.

In summary, our exploration into thyroid surgeries has highlighted the pervasive occurrence of transient hypocalcemia as a common complication, impacting approximately 24% of cases and contributing to heightened morbidity and prolonged hospitalization. Our study, encompassing 132 patients, revealed a consistent predominance of female patients undergoing thyroidectomy, aligning with findings from various global studies. The mean age and demographic distribution of our participants were comparable to those reported in related studies, underscoring the prevalence of older patients and those from rural areas. In addition, our investigation shed light on factors, such as diabetes history, hypertension, body mass index, and serum calcium levels, establishing concordance with analogous studies. The significant incidence of postoperative hypocalcemia, particularly in untreated group, further emphasizes the consistency of our research findings with those from comparable studies, highlighting their collective importance in the broader medical landscape.

## Conclusions

Our study strongly advocates the use of preoperative calcium and vitamin D supplementation for patients undergoing thyroidectomy to combat hypocalcemia after subtotal or total thyroidectomy. The incidence of hypocalcemia was markedly lower in the treatment group compared to the control group. We recommend that all patients undergoing thyroidectomy receive preoperative calcium and vitamin D supplementation to address the issue of hypocalcemia, ultimately reducing the burden of associated morbidities and minimizing prolonged hospitalization.
